# Post-operative pain management modalities employed in clinical trials for adult patients in LMIC; a systematic review

**DOI:** 10.1186/s12871-021-01375-w

**Published:** 2021-05-25

**Authors:** Gauhar Afshan, Robyna Irshad Khan, Aliya Ahmed, Ali Sarfraz Siddiqui, Azhar Rehman, Syed Amir Raza, Rozina Kerai, Khawaja Mustafa

**Affiliations:** 1 Department of Anaesthesiology, 2nd floor Private Wing, Aga Khan University, P.O. Box 3500, Stadium Road, Karachi, 74800 Pakistan; 2 Faculty of Health Sciences, Aga Khan University, P.O. Box 3500, Stadium Road, Karachi, Pakistan

**Keywords:** Post-operative pain, LMIC, Multimodal analgesia

## Abstract

**Background:**

Unrelieved postoperative pain afflicts millions each year in low and middle income countries (LMIC). Despite substantial advances in the study of pain, this area remains neglected. Current systematic review was designed to ascertain the types of clinical trials conducted in LMIC on postoperative pain management modalities over the last decade.

**Methods:**

A comprehensive search was performed in June 2019 on PubMed, Cochrane Library, CINAHL Plus, and Web of Science databases to identify relevant trials on the management of postoperative pain in LMIC. Out of 1450 RCTs, 108 studies were reviewed for quality evidence using structured form of critical appraisal skill program. Total of 51 clinical trials were included after applying inclusion/exclusion criteria.

**Results:**

Results are charted according to the type of surgery. Eleven trials on laparoscopic cholecystectomy used multimodal analgesia including some form of regional analgesia. Different analgesic modalities were studied in 4 trials on thoracotomy, but none used multimodal approach. In 11 trials on laparotomy, multimodal analgesia was employed along with the studied modalities. In 2 trials on hysterectomy, preemptive pregabalin or gabapentin were used for reduction in rescue analgesia. In 13 trials on breast surgical procedures and 10 on orthopaedic surgery, multimodal analgesia was used with some form of regional analgesia.

**Conclusion:**

We found that over the past 10 years, clinical trials for postoperative pain modalities have evolved in LMIC according to the current postoperative pain management guidelines i.e. multi-modal approach with some form of regional analgesia. The current review shows that clinical trials were conducted using multimodal analgesia including but not limited to some form of regional analgesia for postoperative pain in LMIC however this research snapshot (of only three countries) may not exactly reflect the clinical practices in all 47 countries.

Post Operative Pain Management Modalities Employed in Clinical Trials for Adult Patients in LMIC; A Systematic Review.

## Background

“Despite substantial advances in pain research in recent decades, inadequate acute pain control is still more the rule than the exception,” concluded international association for study of pain (IASP) while observing global year against acute pain in 2010–2011. Available data shows a large burden of acute pain in the developed countries, inferring logically, this burden is significantly higher in low and middle-income countries (LMIC). Anaesthesia and related specialties have been reporting the enormity of the burden of pain and suffering in LMIC citing disproportionately limited resources, lack of regulations, and paucity of pain education as the main reasons [1].

Causes of acute pain are numerous, including but not limited to, trauma, burn injury, medical illness, labour, violence, war, man-made and natural disasters, road traffic accidents, and post-operative pain; some being more prevalent in LMIC. Political and social instability in these countries compound the crisis and multiply the acute pain burden manifold [2]. Reported statistics list post-operative pain as the most predominant type of acute pain in LMIC. Absence of efficient basic health care, lack of preventive health, and non-existent disease screening leads to patients presenting with advanced pathology that requires extensive surgical procedures and hence more severe pain [3, 4]. Another reason of poor postoperative pain management is a dearth of strong opioids. Measured in terms of distribution of opioids, only 0.1 metric ton was distributed to LMIC out of a total of 298.5 metric tons of morphine distributed in 2010–2013 in the entire world [5].

Effective postoperative pain management is unquestionably a basic human right. The importance of effective pain relief has long been realized and acute pain services (APS) are operational in majority of the hospitals in the developed world for decades. Big data is available on the subject of postoperative pain management with resultant comprehensive guidelines for the assistance of anaesthesiologists and other physicians managing pain [6–8]. The panel constituted to review literature and formulate acute postoperative pain management guidelines for American Pain Society, American Society for Regional Anesthesia, and American Society of Anesthesiologists (2016) observed that the evidence supports use of multimodal analgesia in most situations though the exact components of multimodal regimen would differ depending upon the patient, setting, and surgical procedure [8]. These guidelines, though quite practical, may not be applicable in their entirety to all health care facilities in the LMIC.

In this age of electronic media, anaesthesiologists, surgeons, and allied health care providers of LMIC are well informed about current recommendations and guidelines but they are hindered by limitation of resources and other factors. Most research, currently available in PubMed, Google and other common search engines, has been conducted in developed countries and their findings might not be acceptable across the world so it is essential to review the published research from LMIC. Current systematic review was designed to chronicle the types of post operative pain management modalities employed in clinical trials for adult patients in LMIC over the last decade.

## Methods

### Search strategy

A systematic literature search was conducted with the assistance of a librarian in PubMed, Cochrane Library, CINAHL Plus, and Web of Science databases to identify all relevant studies on the management of postoperative pain in LMIC. A comprehensive search strategy was developed using a combination of MeSH term “pain, postoperative” with keywords “postoperative pain”, “postoperative pain management”, “postoperative pain relief”, “postoperative analgesia”, “postoperative surgical pain” with suitable Boolean searching [9, 10]. We used the list of LMICs generated by the World Bank which includes 47 countries with a gross national income (GNI) per capita between $1026 and $3995. We included all 47 countries as per the list in our Boolean search [11].

A filter was applied for limiting the search to only human studies published from January 2008 to – June 2019. A total of 2885 studies were extracted and after removing duplicates, 2196 studies were selected. A total of 1450 randomized control trials (RCTs) were found out of 2196 in the pre-specified list of 47 countries.

### Searching and data abstraction

Systematic review team comprised of five anaesthesiologists, one nurse, one biostatistician and one librarian. Two authors in each pair (total of three pairs of five anesthesiologists and one nurse) independently reviewed all potentially eligible 1450 RCTs. A total of 1342 were excluded after screening titles, reviewing abstracts and considering the objectives.

**Fig. 1:**
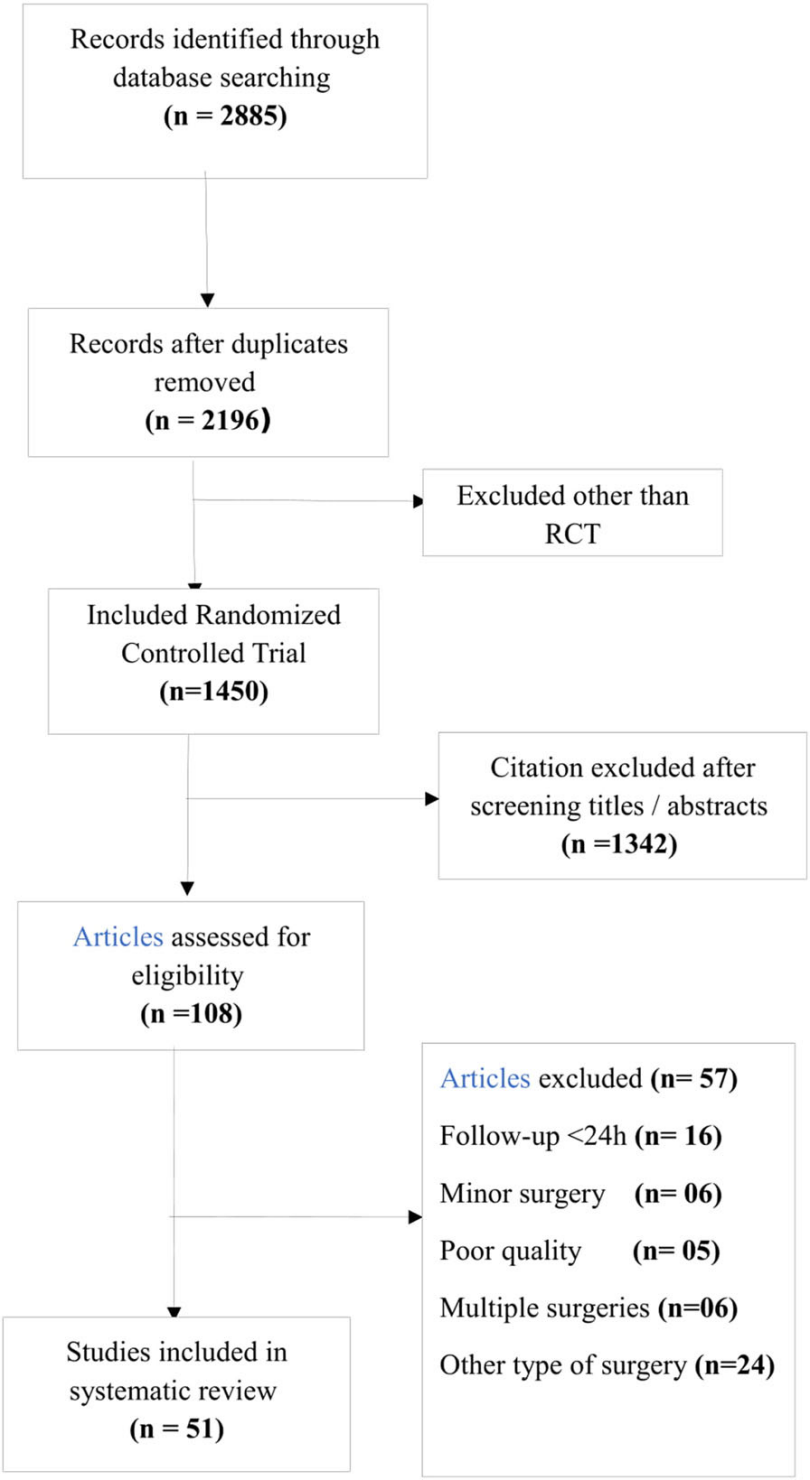
PRISMA Flow Chart

Full-text versions of 108 RCTs were reviewed using 11 questions, based on the structured form of CASP (critical appraisal skill program) by the same reviewers. Disagreements were resolved through open discussion and consensus. Finally, inclusion/exclusion criteria were applied. Common surgical procedures i.e. laparoscopic cholecystectomy, mastectomy, total abdominal hysterectomy, laparotomy, and orthopedic extremity surgery were included. Studies were excluded if post- operative pain management was provided for less than 24 h and/or no rescue analgesia was planned. Finally we selected RCTs fulfilling the inclusion & exclusion criteria for the review as mentioned in the PRISMA diagram (Fig. 1). Patient characteristics (age and gender), study characteristics (name of the country and type of surgery), information on pain severity, pain measurement scale, types of different pain modalities (used), rescue analgesia (used), and duration of postoperative pain control were recorded in a structured format (Table 1). Systematic review team ensured that important studies were not missed; however publication bias is a possibility despite the due diligence observed while conducting the literature search.

**Table 1:** Characteristics of the published randomized controlled trails presented with respect to type of surgery

Authors	Country	Age Range	Gender	N	Groups	N	Modalities Studied	Pain Assessment Tool	Modalities used	Duration of post-operative Pain studied	Rescue Analgesia
Bhatia et al., (2014) [12]	India	18–60	Both	60	3	20	Ultrasound-guided posterior transversus abdominis plane (TAP) block with 15 mL of 0.375% ropivacaine	VAS (Visual Analogue Scale)	Single	24 h	Tramadol
						20	Ultrasound-guided subcostal TAP block with 15 mL of 0.375% ropivacaine				
						20	Control				
Shukla et al.,(2015) [13]	India	18–60	Both	120	3	40	Intraperitoneal bupivacaine 50 ml 0.25% + 5 ml Normal saline	VAS	Single	24 h	Diclofenac
						40	Bupivacaine 50 ml 0.25% + tramadol 1 mg/kg (diluted in 5 ml NS)				
						40	Bupivacaine 50 ml 0.25% + dexmedetomidine 1 μg/kg, (diluted in 5 ml NS)				
Sinha et al., (2016) [14]	India	18–65	Both	60	2	30	TAP block with 0.25% Bupivacaine	VAS	Single	24 h	Diclofenac
						30	TAP block with 0.375% Ropivacaine				
Kamhawy et al., (2017) [15]	Egypt	21–60	Both	46	2	23	Unilateral subcostal TAP block	VAS	Multi	24 h	PCA Morphine
						23	Unilateral thoracic paravertebral block				
Saxena et al., (2016) [16]	India	18–70	Both	80	2	40	Local anesthetic infiltration of surgical incision	NRS (Numerical rating score)	Multi	24 h	Fentanyl
						40	Bilateral rectus sheath and right TAP blocks				
Ali et al., (2012) [17]	Pakistan	35–65	Both	60	2	30	Oral pregablin 150 mg	VAS	Single	24 h	Nalbuphine
						30	Oral celecoxib 200 mg				
Suseela et al., (2018) [18]	India	20–65	Both	80	2	40	Bilateral ultrasound-guided subcostal TAP block with 20 mL of 0.25% bupivacaine	NRS	Multi	24 h	Tramadol Diclofenac
						40	Port-site infiltration with 20 mL of 0.5% bupivacaine				
Anand et al.,(2017) [19]	India	20–60	Both	60	2	30	Alprazolam	VAS	Multi	24 h	Tramadol
						30	Pregabalin				
Pasha et al., (2018) [20]	Nowshera	18–65	Both	90	2	45	Gabapentin	VAS	Multi	24 h	None
						45	Placebo				
Khan et al., (2018) [21]	pakistan	18–60	Both	126	2	63	Posterior TAP block with 0.375% bupivacaine	NRS	Multi	24 h	Tramadol
						63	subcostal TAP block with 0.375% bupivacaine				
Jain et al., (2018) [22]	India	20–60	Both	60	2	30	Intraperitoneal Saline with 500 ml	NRS	single	24 h	Tramadol
						30	Intraperitoneal Bupivacaine with 20 ml of 0.5% (100 mg)				
Amr et al., (2010) [23]	Egypt	18+	Both	40	2	20	Preincisional thoracic epidural analgesia (TEA)	VAS	Single	24 h	None
						20	End of surgery TEA				
Khalil et al., (2017) [24]	Egypt	20–60	Both	40	2	20	Ultrasound-guided serratus anterior plane block	VAS	Multi	24 h	Morphine
						20	TEA				
Dutta et al., (2017) [25]	India	18–70	Both	30	2	15	Bolus of 15 mL of 0.75% ropivacaine over 3 to 5 min, followed by an infusion of 0.2% ropivacaine at 0.1 ml/kg/hour	VAS	Multi	24 h	Morphine
						15	15 ml of 0.75% ropivacaine plus dexmedetomidine,1 mg/kg bolus over 3 to 5 minutesfollowed by an infusion of 0.2% ropivacaine plus 0.2 mg/kg/hour of dexmedetomidine at 0.1 ml/kg/hour.				
Biswas et al., (2016) [26]	India	18–60	Both	60	2	30	Epidural: 7.5 ml bolus of 0.125% Bupivacaine with 50 μg Fentanyl	VAS/ FPORS	Muti	24 h	Tramadol
						30	Paravertebral 15 ml bolus of 0.125% Bupivacaine with 50 μg Fentanyl				
Bakshi et al., (2016) [27]	India	18–75	Female	71	2	36	Rectus sheath block with 0.25% bupivacaine	NRS	Multi	48 h	Morphine
						35	Rectus sheath block with normal saline				
Dhanapal et al., (2014) [28]	India	18+	Both	94	2	47	Wound infusion with Bupivacaine	VAS	Single	48 h	Morphine
						47	Wound infusion with N saline				
Wahba et al., (2014) [29]	Egypt	59–75	Both	44	2	22	Thoracic epidural infusion	VRS	Multi	48 h	Morphine (PCA)
						22	Bilateral TAP block infusion with catheter				
Sethi et al., (2014) [30]	India	18–45	Both	100	2	50	Patient controlled epidural analgesia (PCEA) with Ketamine + Morphine	VAS	Multiple	48 h	Diclofenac
						50	PCEA Morphine				
Moawad et al., (2014) [31]	Egypt	20–60	Both	100	2	50	PCEA Bupivacaine + Fentanyl	NRS	Multiple	24 h	Fentanyl
						50	Intravenous patient controlled analgesia (PCA) with Fentanyl				
Patil et al., (2018) [32]	India	18–65	Female	60	2	30	Thoracic epidural infusion with 0.125% Ropivacaine and Fentanyl 0.125% Bupivacaine and Fentanyl	VAS	Multi	24 h	Tramadol
						30					
Bharti et al., (2018) [33]	India	Adult patients	Both	40	2	20	50 μg Dexmedetomidine with 10 ml of 0.125% Bupivacaine in thoracic epidural	VAS	Multi	24 h	Diclofenac
						20	50 μg Fentanyl with 10 ml 0.125% Bupivacaine in thoracic epidural				
Alvi et al., (2017) [34]	Pakistan	18–50	Both	200	2	100	TAP block	NRS	Single	24 h	None
						100	Placebo block				
Patel et al., (2018) [35]	India	20 to 65	Both	60	2	30	TAP block with Ropivacaine (0.5%) 20 ml	VAS	Multi	24 h	Diclofenac
						30	Spinal anaesthesia				
Mishra et al., (2018) [36]	India	18–60	Both	60	2	30	Thoracic paravertebral block (20 mL 0.25% bupivacaine)	VAS	Multi	24 h	Tramadol
						30	IV PCA with fentanyl				
Bharti et al., (2011) [37]	India	18–60	Both	40	2	20	TAP Block with 20 mL of 0.25% bupivacaine	VAS	Single	24 h	Morphine
						20	TAP Block with Saline				
Chotton et al., (2014) [38]	India	18–60	Female	90	2	45	Pregabalin 150 mg	VAS	Multi	24 h	Ketorolac
						45	Placebo				
Badawy et al., (2015) [39]	Egypt	40–70	Female	60	3	20 20 20	Oral gabapentin 800 mg Gabapentin 800 mg + Dexamethasone 8 mg Placebo	VAS	Multi	24 h	Meperidine
Bashandy et al., (2015) [40]	Egypt	Adult	Female	120	2	60 60	Ultrasound-guided Pecs block Control	VAS	Single	24 h	Morphine
Hetta et al., (2016) [41]	Egypt	Adult	Female	111	4	28 27 26 30	Pregabalin 75 mg Pregabalin 150 mg Pregabalin 300 mg Placebo Capsule	VAS	Single	24 h	Morphine
Kasimahanti et al.,(2016) [42]	India	18–60	Female	58	2	28 30	Single-level, unilateral ultrasound-guided TPVB at T4 level using 0.3 mL/kg of 0.5% ropivacaine Double-level, unilateral ultrasound-guided TPVB at T2 and T5 level using a total volume of 0.3 mL/kg of 0.5% ropivacaine	NRS	Single	24 h	Diclofenac
Kulhari et al., (2016) [43]	India	18–65	Female	40	2	20	TPVB with ropivacaine 0.5%, 25 ml,	VAS	Single	24 h	Morphine
						20	PECS II block with ropivacaine 0.5%				
Gupta et al., (2017) [44]	India	18–65	Female	50	2	25	Paravertebral block	VAS	Multi	72 h	PCA Morphine
						25	Serratus Plane block				
Bhuvaneswari et al.,(2012) [45]	India	Adult	Female	48	4	12	Paravertebral block with 0.25% bupivacaine with epinephrine 5 mcg/ ml	VAS	Single	24 h	Morphine
						12	Paravertebral block with 0.25% bupivacaine + epinephrine 5 mcg/ml with 2 mcg/ml fentanyl				
						12	Paravertebral block with 0.5% bupivacaine + epinephrine 5 mcg/ml or isotonic saline				
						12	Noraml Salin				
Mahran et al., (2015) [46]	Egypt	18–65	Female	90	3	30	Pregabalin 150 mg oral	VAS	Multi	24 h	Morphine
						30	Placebo capsule (oral) and 0.5 mg/kg ketamine IV				
						30	Placebo				
Kundra et al., (2013) [47]	India	Adult	Female	120	2	60	Paravertebral block	VAS	Multi	24 h	No rescue analgesia
						60	Interpleural block				
M. Neetu et al., (2018) [48]	India	18–70	Female	60	2	30	PECS block	VAS	Multi	24 h	None
						30	Placebo				
Mukherjee et al., (2018) [49]	India	35–60	female	88	2	44	Ropivacaine (0.5%)	VAS	single	48 h	Diclofenac
						44	Dexmedetomidine 1 μ/kg				
Megha et al., (2018) [50]	India	18–60	Female	47	2	23	Paravertebral block with 20 ml bupivacaine 0.25% with morphine 3 mg	NRS	Multi	24 h	Diclofenac
						24	Dexmedetomidine 1 μg/kg				
Manzoor et al., (2018) [51]	India	18–70	Female	60	2	30	PECS I block with 30 ml 0.25% bupivacaine	VAS	single	24 h	Diclofenac
						30	PECS I block with 10 ml 0.25% bupivacaine with dexmedetomidine				
Kumar et al., (2018) [52]	India	?	Female	50	2	25	Opioids and non-steroidal anti-inflammatory drug	VAS	Multi	24 h	Tramadol
						25	PECS I with 0.25% bupivacaine				
Bharti et al., (2015) [53]	India	20–60	Both	54	2	27	Supraclavicular block with 1 μg/kg of Dexmedetomidine along with equal volumes of 0.75% ropivacaine and 2% lidocaine with adrenaline.	VAS	Single	24 h	Tramadol Diclofenac
						27	0.75% Ropivacaine and 2% Lidocaine with adrenaline (1:2,00,000)				
Kumar et al., (2014) [54]	India	18–60	Both	30	2	15	Ultrasound-guided stellate ganglion block with 2% Lidocaine	VAS	Single	24 h	PCIA Used [No Rescue]
						15	Ultrasound-guided stellate ganglion block with 0.9% Saline				
Mullaji et al., (2010) [55]	India	50–80	Both	40	2	20	Combined spinal epidural + local anesthetic infiltration	VAS	Single	24 h	None
						20	Combined spinal epidural with no local anesthetic infiltration				
Khanna et al., (2017) [56]	India	40–60	Both	90	3	30	Epidural with ropivacaine 0.1%	VAS	Multi	36 h	PCEA
						30	Epidural with ropivacaine 0.1% with fentanyl 2.5μg/mL				
						30	Epidural with ropivacaine 0.0625% with fentanyl 2.5μg/mL				
Anis et al., (2011) [57]	Egypt	18–60	Both	60	3	20	Lumbar plexus block with 15 ml bupivacaine 0.25% + clonidine	VAS	Single	24 h	Morphine
						20	Lumbar plexus block with bupivacaine 0.25% + clonidine				
						20	No Block				
Sawhney et al., (2015) [58]	India	Adult	Both	100	4	25	Epidural with 0.2% Ropivacaine	VAS	Single	24 h	Tramadol
						25	Epidural with0.1% Ropivacaine+Fentanyl 2 μg/ml				
						25	Epidural with 0.2% Bupivacaine				
						25	Epidural with Bupivacaine 0.1% with Fentanyl 2 μg/ml.				
Trabelsi et al., (2017) [59]	Tunisia	> 18	Both	60	2	30	Suprascapular block + supraclavicular block	VAS	Multi	24 h	Morphine
						30	Interscalene block				
Meghana et al., (2017) [60]	India	20–65	Both	70	2	35	0.125% bupivacaine and 2 mg/ml fentanyl epidural infusion	NRS	Single	48 h	Tramadol
						35	0.2% ropivacaine and 2 mg/ml fentanyl as epidural infusion				
Thakur et al., (2015) [61]	India	18–60	Both	67	3	22	Axillary brachial plexus block with bupivacine, lignocaine, adrenaline and buprenorphine + IM placebo	VAS	Single	24 h	Diclofenac
						23	Axillary brachial plexus block with bupivacine, lignocaine, adrenaline and placebo + IM buprenorphine				
						22	Axillary brachial plexus block with bupivacine, lignocaine, adrenaline and placebo + IM placebo				
Mostafa et al., (2018) [62]	Egypt	50–70	Both	60	2	30	Levobupivacaine 0.125%	VAS	Multi	24 h	None
						30	IV fentanyl 20 μg/ml				

## Results

A total of 51 RCTs were included for the review. It is worth noting that only three countries among the list of LMIC have published RCTs fulfilling the predetermined inclusion & exclusion criteria. The review results were charted according to the type of surgery.

### Laparoscopic cholecystectomy

Total of 11 RCTs [12–22] were included in the review (Table 1). These studies collectively described 842 patients of both genders with age range of 18–70 years. Majority used some form of regional analgesia. Transversus abdominis Plane (TAP) block comparing conventional and subcostal approaches was used in two RCTs [12, 21], and TAP block comparing two local anesthetics (LA) in one [14]. TAP block was compared with LA infiltration of incisional wounds in one RCT [18]. Intraperitoneal infiltration of LA comparing different drugs was used in two trials [13, 15] while intraperitoneal LA infiltration was compared with placebo in one [22]. One study compared LA infiltration of incisional wounds with abdominal plane blocks [16]. Oral Pregabalin was compared with Celecoxib in one trial [17], oral Pregabalin with Alprazolam in one [19] and Gabapentine with placebo in one [20]. All trials used multimodal analgesia for pain management, while comparing one or more modalities.

### Thoracotomy

Total of 4 [23–26] RCTs were included for postoperative pain management following thoracotomies (Table 1). These trials collectively described 170 patients of both genders with age range of 18–70 years [23–26]. Various analgesic modalities have been studied including continuous thoracic epidural analgesia, serratus anterior plane block (SAPB), and continuous paravertebral block. None of the trials used multi-modal approach. Three RCTs studied regional blocks with rescue analgesia [24–26] while one RCT studied continuous thoracic epidural analgesia without rescue analgesia [23]. Continuous Paravertebral dexmedetomidine was also used in one trial to decrease the intraoperative anaesthetic requirement and post-thoracotomy pain syndrome [25].

### Laparotomy / Other Abdominal Surgery

A total of 11 RCTs [27–37] were identified related to laparotomies and other open abdominal surgeries including a total number of 869 patients aged between 18 and 75 years (Table 1). Nine trials included patients of both genders, while two included females only. Various analgesic modalities were studied for postoperative pain relief following laparotomies and open abdominal procedures. One trial employed rectus sheath block comparing 0.25% bupivacaine with saline [27]. Another trial studied continuous wound infusion comparing bupivacaine with saline [28]. Thoracic epidural was used in five RCTs [29–33]. In one trial it was compared with continuous infusion through bilateral TAP block [68]. Another trial had compared patient controlled epidural analgesia (PCEA) with a combination of morphine and ketamine with morphine alone [30]. One trial compared bupivacaine-fentanyl PCEA with fentanyl patient controlled intravenous analgesia (PCIA) [31]. Another compared bupivacaine and fentanyl with ropivacaine and fentanyl infusion through thoracic epidural [32]. Yet another trial employing thoracic epidural compared dexmedetomidine and fentanyl as adjuncts to local anaesthetic [33]. TAP block was compared with placebo (normal saline) in three RCTs [34, 35, 37]. Thoracic paravertebral block was compared with IV PCA in another trial [45]. Multimodal analgesia was employed in all trials along with the modalities being studied. Morphine, pethidine, or diclofenac was used for rescue analgesia in these RCTs.

### Hysterectomy

In 2 RCTs [38, 39] performed on patients undergoing TAH, 150 patients were included, aged 18–70 years (Table 1). Preoperative pregabalin was compared with placebo in one of the trials, while gabapentin with or without dexamethasone was compared with placebo in the other. The effect of these drugs on postoperative analgesic consumption was studied. Rescue analgesia was provided with ketorolac [38] or pethidine [39].

### Breast Cancer surgery

A total of 13 RCTs [40–52] were included. In 11 trials, multimodal analgesia was used with some form of regional analgesia (Table 1). In one trial [41], oral pregabalin was used pre-operatively for reducing postoperative pain and morphine consumption in patients undergoing mastectomy. In another trail [46], preoperative oral pregabalin (150 mg) was compared with intravenous ketamine (0.5 mg.kg-1) at induction of anaesthesia and showed reduction in postoperative opioid consumption without increasing sedation and with a good safety profile. In seven RCTs, thoracic paravertebral block (TPVB) was used for intra and post-operative pain management. In one trial [42], single level TPVB was compared with block at two different levels. Bupivacaine was used with epinephrine in one trial [45], while one used ropivacaine with dexmedetomidine [49] as adjuvants with better post-operative pain relief. One trial compared morphine and dexmedetomidine with bupivacaine in TPVB [50]. In one trial, TPVB was compared with intrapleural block [47]. In two trials, TPVB was compared with newer blocks like PECS II block [43] and serratus plane block [44]. In two RCTs [40, 48] ultrasound-guided PECS I & II blocks were used for pain management. In one trial [42], bupivacaine 0.25% was used with or without dexmedetomidine for PECS I & II blocks. In one study [52], ultrasound guided PECS I & II blocks were compared with intravenous opioids and NSAIDS.

### Orthopedic procedures

A total of 10 RCTs [53–62] fulfilled the inclusion criteria. Four RCTs included upper limb procedures (Table 1). In these trials, peripheral nerve blocks (supraclavicular, stellate ganglion, supraclavicular and axillary nerve blocks) were compared either with different concentrations of local anaesthetic agents or with other modalities such as intramuscular narcotics [53, 54, 59, 61]. In lower limb procedures, 2 RCTs were performed on total knee arthroplasty and the modalities used were sub-arachnoid block, lumbar epidural and intra articular infiltration of local anaesthetic drugs. Different local anaesthetic agents were compared in different concentrations [55, 56] Two RCTs were done on hip surgery [57, 62] and two were on generalized lower limb procedures [58, 60]. Lumbar plexus block, lumbar epidural, fascia iliaca compartment block and intravenous narcotics were compared.

## Discussion

Ineffective pain management in the postoperative period leads to untoward consequences like slower recovery and increased cost of care. Optimal pain management modalities enable earlier mobilization and ease of performing physical therapy with resultant early functional recovery. Recent decades have seen a surge of research directed towards improvement in the quality of postoperative pain relief with special focus on procedure specific pain management. Bulk of this research has originated from the developed world.

Systematic reviews are now being carried out in health care systems to get the best evidence for decision making and to subsequently include the researched modality/intervention in the clinical practice. Two main purposes of a systematic review are to establish the extent to which existing research has progressed toward explaining a problem, and to clarify the extent to which this evidence explains a new or existing question. The purpose of this systematic review is to deliver a meticulous summary of all the available RCTs performed in LMIC over the last decade for the management of postoperative pain in adult patients, to scrutinize the types of modalities being used in LMIC for postoperative pain relief, and to compare these modalities with those being used in the developed world.

The PROSPECT is an international collaboration of anaesthesiologists and surgeons. The PROSPECT aims to provide healthcare professionals with practical procedure-specific pain management recommendations formulated in a way that facilitates clinical decision-making across all the stages of the perioperative period [63]. For postoperative pain management for laparoscopic cholecystectomy procedure, PROSPECT [64] recommends multimodal analgesia including wound infiltration with long acting local anaesthetic (LA), intraperitoneal infiltration of LA or both, paracetamol, COX-2 selective inhibitors, NSAIDs, and opioids for rescue analgesia. Four out of 11 RCTs from LMIC used regional blocks, which are neither recommended, nor not-recommended in PROSPECT. However, that can be due to PROSPECT recommendations being formulated in 2005 while use of abdominal wall blocks is rather a recent phenomenon. Intraperitoneal infiltration of LA was studied in three instances. One trial compared LA infiltration of incisional wounds with abdominal plane blocks. Oral Pregabalin, Cox-2 inhibitor, and Gabapentin were also studied. Majority [five] RCTs used TAP blocks for the study group, intraperitoneal infiltration with LA in three, and gabapentinoids in three. Usual care or control groups received either TAP block at a different level than the study group [subcostal vs. conventional], different drugs or different concentrations of the same drugs. LA infiltration of the surgical wounds was employed in two control groups while celecoxib and alprazolam were used for two. One trial used placebo for control group in place of the studied modality. All trials used multimodal analgesia for pain management overall, which is according to the international recommendations.

Thoracotomy is considered one of the most painful surgical procedures. Inadequate pain relief after thoracotomy can result in postoperative pulmonary complications. Considering multifactorial nature of thoracotomy, a single modality cannot provide adequate pain control. The management of pain after thoracotomy requires a multimodal approach incorporating regional and systemic analgesia to targets multiple sites [65]. Regional analgesia is highly recommended with non-steroidal anti-inflammatory drugs (NSAIDs), paracetamol, opioids and other adjuvants for the pain following thoracotomy. Analgesic effect of paracetamol with NSAIDs is additive. None of the RCTs used NSAIDs and paracetomol for post thoracotomy pain.

Continuous thoracic epidural analgesia is recommended by PROSPECT for postoperative pain management following laparotomy, ensuring an appropriate level according to the site of incision [63]. A combination of local anaesthetic agent and an opioid for the epidural infusion has better analgesic efficacy compared to either agent alone. When the patient does not receive an epidural due to contra-indication or lack of feasibility, strong opioids using intravenous patient controlled analgesia (IV PCA) are recommended for high intensity pain as part of a multimodal regime. Multimodal analgesia in such cases may include non-steroidal anti-inflammatory drugs, paracetamol and intravenous lignocaine. Pre-peritoneal infusion of local anesthetic is recommended in patients who have not received an epidural. The RCTs s performed on postoperative pain relief in LMIC have employed multimodal analgesia in all cases as recommended for these procedures. Thoracic epidural was used in five of the 11 trials, while PCA was used in two. Majority RCTs [five] used thoracic epidural followed by TAP block [three]. In the case of usual care or control groups, thoracic epidural was employed but using either a different drug or a different concentration, while IV PCA was used for two control groups. Placebo was used in the control groups in three studies, replacing the studied modality. Abdominal wall blocks were employed in five studies, which are not part of the PROSPECT recommendations. As pointed out above, abdominal wall blocks have come in vogue after the PROSPECT recommendations. Since multiple and varied pain relieving modalities have been employed and compared in different studies, it is difficult to compare the results with the current recommendations.

Multimodal analgesia is recommended for postoperative pain relief following total abdominal hysterectomy (TAH). For severe pain, PROSPECT recommends strong opioids using PCA along with NSAIDs such as Diclofenac. Opioids can be administered as a continuous infusion, when PCA is not feasible. Weak opioids can be substituted along with paracetamol and NSAIDs when pain decreases to moderate intensity [66]. Though epidural analgesia is not recommended for routine use, it is considered useful for high-risk patients undergoing TAH. In both the RCTs conducted in LMIC on post-hysterectomy pain, multimodal analgesia was not employed, rather pre-emptive analgesic effect of gabapentin (plus dexamethasone) or pregabalin was studied on consumption of a single postoperative analgesic agent (ketorolac [38] or pethidine [39]. Both used placebo for usual care groups.

A multimodal approach has been recommended for perioperative pain management in major breast cancer surgery. A successful multimodal approach requires coordination between surgical, anaesthesia, and nursing staff throughout perioperative period. Recent recommendations [67] are to use antiepileptic medication or gabapentinoids (gabapentin or pregabalin), paracetamol, and regional nerve blocks (paravertebral blocks, PEC blocks, or thoracic epidural injection), wound infiltration with LA at the end, NSAIDs, and intermittent short-acting opioids. This regimen should be continued for up to 1 week after surgery. Other classes of medications can also be used such as, intravenous lignocaine, N-methyl-D-aspartate (NMDA) antagonists such as ketamine and magnesium, alpha-2-adrenergic antagonists clonidine and dexmedetomidine. Glucocorticoids such as dexamethasone have been used to minimize postoperative pain, nausea and vomiting.

PROSPECT recommendations for non-cosmetic major breast surgery [68] include paravertebral block, gabapentinoids, COX-2-selective inhibitors, paracetamol, IV dexamethasone, intercostal nerve block plus other regional techniques (TPVB), NSAIDs, strong opioids, (for high intensity pain) or weak opioids for moderate to low intensity pain, paracetamol alone or in combination with other non-opioid analgesics for low to moderate intensity pain. Majority of the RCTs [seven] employed thoracic paravertebral blocks, followed by PECS I and II block [four]. Though regional techniques were employed, there was a gap in comparison to the recent recommendations, such as preoperative use of antiepileptic medication or gabapentinoids, paracetamol and, intraoperative wound infiltration with LA, NSAIDs, and intermittent short-acting opioids. Usual care or control groups used different drugs or different concentrations of the drug for TPVB and PECS I and II blocks. In LMIC, incidence of breast cancer is rising and increasing number of patients are undergoing these procedures. Healthcare teams hence are required to develop and follow multimodal pain management protocols as per recent recommendations to provide quality care to their patients. Multimodal preventive analgesia regimen needs to be followed in patients scheduled for major breast cancer surgery.

Moderate to severe pain is not uncommon after orthopedic procedures, especially after joint replacement surgeries. If not adequately controlled, there is a high probability of developing persistent post-surgical pain. Two commonly performed procedures in the lower limb are total knee arthroplasty (TKA) and hip replacement surgery. In RCTs carried out in LMIC, the modalities used for TKA were local anaesthetic infiltration in joint space, lumbar epidural, combined spinal epidural, and lumbar plexus block. According to the PROSPECT recommendations [68] for TKA, peripheral neural block is strongly recommended for best post-operative pain management. Epidural block is only recommended for patients having increased risk of cardio-pulmonary complications and in those cases where general anaesthesia is contraindicated due to increased risk of morbidity; otherwise epidural is not recommended for post-operative analgesia after TKA. Intra-articular infiltration of local anaesthetics is also not recommended because of inconsistent evidence. Similarly ASA (American Society of Anesthesiologists) strongly recommends the use of peripheral nerve blocks, either continuous or single shot, after TKA and hip surgeries [69]. Hence in LMIC, the post-operative pain management practices for lower limb surgeries are not according to the evidence based recommended methods, which is probably due to lack of expertise in performing peripheral nerve blocks, lack of knowledge, or due to a large patient volume.

On the contrary, for upper limb and shoulder surgeries the studies done in LMIC have shown that peripheral nerve blocks were used for post-operative pain management. ASA also strongly recommends peripheral nerve blocks for upper extremities and shoulder surgery. However there is no recommendation by PROSPECT for upper limb surgeries as yet. Hence the pain management strategies for upper limb surgeries in LMIC seem to be consistent with the current practice of the developed countries.

This systematic review addresses the post-operative pain management in LMIC for the first time. A potential limitation of this review is the inclusion of last 10 years studies with a wide range of clinical outcomes. The included studies were conducted in only three LMICs out of total 47 listed. It is difficult to estimate the direct cost of postoperative pain in LMICs in included studies due to non- availability of the data regarding resumption of routine functions. Though the review shows a congruence of RCTs being carried out in the LMICs with internationally available recommendations and guidelines in majority of the instances, it is pertinent to realize that clinical practices on the ground may not reflect this. The findings of this review should be interpreted cautiously as majority of RCTs are small. This indeed is another limitation of the review. Placebo was used in four RCTs for the control groups, replacing study drug/intervention. Although there were other analgesia options in the multimodal regimen being used to treat pain, use of placebo is outdated and not encouraged for pain research. This systemic review, based on RCTs on postoperative pain management in LMICs, identified numerous research gaps in the included small sample sized low-quality studies. Authors believe that there is an urgent need to conduct research on practice gaps regarding the use of cost-effective evidence-based management of postoperative pain in LMICs.

## Conclusion

Three billion people live in LMICs out of a total world population of 7.53 billion. Scientific literature is very scant coming from the part of the world housing nearly half its population. Guidelines and recommendations are formulated based on research carried out entirely in the other half, yet LMICs try to follow them. The current review shows the same trend. Multimodal analgesia is being used for majority of the procedures; while use of regional analgesia as part of multi-modal analgesia was common however this research snapshot (of only three countries) may not exactly reflect the clinical practices in all 47 countries.

Systematic reviews do not merely determine what is being done but also identify and document knowledge gaps in the literature. These gaps then can be used to shape future research agendas in the LMICs related to any question, for example post-operative pain. It is essential to realize that improved health care practices require evidence based research carried out in LMIC to guide development of relevant and contextual standards of care. The authors strongly recommend the conduct of more RTCs in LMIC based on the available resources for postoperative pain management rather than conducting them in accordance with international guidelines of developed countries.

## Data Availability

All data generated or analysed during this study are included in this published article.

## Abbreviations

LMIC: low and middle-income countries
GNI: gross national income
RCTs: randomized control trials
CASP: critical appraisal skill program
PCEA: patient controlled epidural analgesia
PCIA: patient controlled intravenous analgesia
TAH: total abdominal hysterectomy
APS: acute pain services
TKA: total knee arthroplasty
ASA: American Society of Anesthesiologists
TAP: transversus abdominis plane
SAPB: serratus anterior plane block
TPVB: thoracic paravertebral block
LA: local anaesthetic
NSAID: non -steroidal anti-inflammatory drugs
NMDA: N-methyl-D-aspartate
